# Hydatids in the Bones of the Pelvis

**Published:** 1842-10-29

**Authors:** 


***Hydatids in the Bones of the Pelvis.***—A labourer, aged 42 years, had suffered in his youth from repeated attacks of swelling of the cervical and axillary glands. For five years he had been affected by syphilis, suffering under gonorrhœal sores on the glans, and buboes. In the year 1833, he experienced periodical pains in the left hip, which were at first supposed to be rheumatic. These increased to such a degree that he could not bear the least pressure on the part, and were especially severe at night, in the loins and left hip. He was admitted into the hospital at Vienna in March, 1834. At this time the appearance of the patient betokened severe suffering: the pulse was quick; the appetite lost; and nights sleepless. The left hip was swollen, and several whitish immoveable swellings, of the size of a chicken's egg, were perceived in this situation. The thigh was flexed upon the pelvis, and any attempt to straighten it was attended with a great increase of pain, as was also every endeavor of the patient to move himself, or turn in bed. After his admission, cold applications were applied, with occasional abstraction of blood by leeches, and three moxas, which had the effect of reducing the swelling, and diminishing the pain a little; but very little increase in the mobility of the limb followed. Subsequently diarrhœa, with hectic, followed, and the patient sunk in May, 1834.

On post-mortem examination, the left os innominatum was found swollen and soft. The upper part of the ilium was degenerated into an oblong osseo-fibrous sac, which was filled with fragments of bone and hydatids, varying in size from a hemp-seed to a walnut. Even the cells in these fragments of bone were stuffed with hydatids. Connected with the pelvic bones were projections of the size of a walnut, or hen's egg, which proved to be cavities formed by the expanded walls of the bone, and the thickened periosteum, or bounded by the latter alone. Some of these projected into the cavity of the pelvis, forming bags, communicating with the cancellous texture of the bone by larger or smaller apertures. They contained irregular plates of bone, of various sizes, and hydatids, with firm transparent walls, differing in size from a scarcely perceptible grain of sand, to a hazel-nut, which lay loose between the fragments of bone, sometimes one hydatid occupying an entire cell; at others, several were grouped together, and connected with the fine membrane which lined the cavity of the bone. The hip-joint was also swollen, and a section of the capsular membrane showed that hydatids were contained within it.

This case, taken in connection with others of a similar nature, related by Fricke and Cullerier, renders it probable, in the author's opinion, that the disease was connected with the syphilitic taint which existed in the system.—*London Medical Gazette, Sept.* 23, 1842, *from Oppenheim Zeitschrift für die Gesammte Medicin.*

